# How Far Can We Hear with the Telephone?

**Published:** 1879-12

**Authors:** 


### Selections.

### HOW FAR CAN WE HEAR WITH THE TELEPHONE?

This is a question frequently asked, but we believe has not yet been definitely settled. The longest distance that we have seen mentioned is given in the item below, namely, two thousand miles. But perhaps Mr. Edison has had more extended experiences. If so we should be glad if he would let our readers know.

An exchange states that Mr. Robert A. Packer, superintendent of the Pennsylvania Railroad, is at present hunting with a party of gentlemen in Nebraska. A few days ago he for two hours conversed pleasantly with his wife and friends at Sayre, Pa., his brother at Mauch Chunk, Pa., and friends along the line. The medium was the railroad and Western Union Telegraph wires and Edison's telephone. At the office in Bethelehem, Pa., connection was made with the Easton and Amboy wire, and at Perth Amboy with a Western Union wire, and thence to Chicago and North Bend, Nebraska, where the party are. The distance was about two thousand miles, and every whisper was audible.—*Scientific American.*

